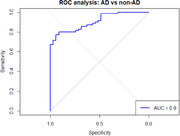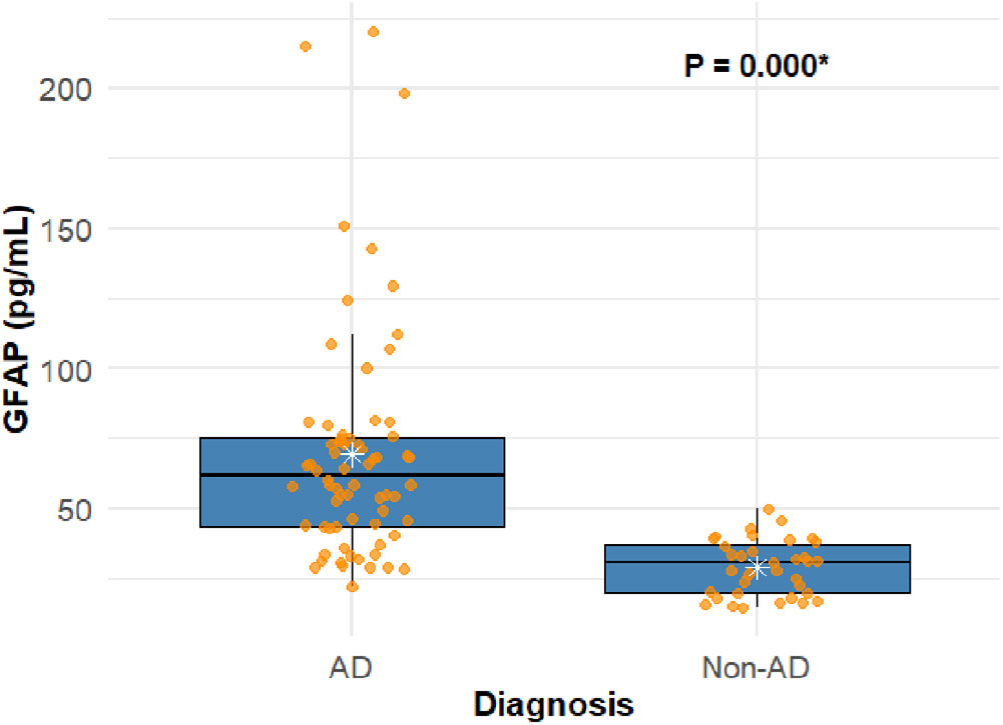# Real‐Life plasma GFAP Application using Chemiluminescent Microparticle Immunoassay in an expanded Cohort

**DOI:** 10.1002/alz70856_104878

**Published:** 2026-01-08

**Authors:** Eduard Bargay Pizarro, Daniel Morell García, Susana Tarongi Sanchez, Ana García Martin, Marian Vives Crook, Margalida Sastre Mesquida, Lara Nuñez Santos, María Santés Bertó, Guillermo Amer Ferrer

**Affiliations:** ^1^ IdISBa, Palma, Spain; ^2^ Son Espases University Hospital, Palma, Spain

## Abstract

**Background:**

The AA 2024 revised diagnostic criteria for Alzheimer's disease (AD) included plasma glial fibrillary acidic protein (pGFAP) as a key blood biomarker of inflammation in AD. This underscores the need for real‐world validation and targeted studies to better understand its clinical utility and application. We previously validated pGFAP's diagnostic utility for AD using a low‐cost chemiluminescent microparticle immunoassay (CMIA; Alinity i, Abbott, US) (AAIC2024). Now, we aim to assess its practical application in a larger, real‐world memory clinic cohort.

**Method:**

From February 2023 to May 2024, core AD CSF biomarkers were obtained as the diagnostic workup of 109 subjects. Mixed ratios Aß42/40 and/or *p*‐tau181/Aß42 were used for AD diagnosis. pGFAP was simultaneously determined using CMIA. Four patients were excluded due to confounding factors increasing pGFAP levels. Clinical and neuroimage evaluations were collected.

**Result:**

We analyzed 105 patients (age 74 years [IQR 70‐78], female 60%, pGFAP 47 pg/mL [IQR 37‐75], MMSE 25.5 [IQR 23.0‐27.0]). Seventy patients were classified as AD (40 MCI, 30 dementia) and 35 as non‐AD (FTD, LBD, vascular and other), with no significant differences in age, sex or MMSE. pGFAP was higher in the AD group (62 pg/mL [IQR 44, 75] vs 31 pg/mL [IQR 20, 37], *p* <0.001). Age, but not sex, was associated with elevated pGFAP (*p* = 0.030). Results remained significant after adjusting for covariates. In this real‐life cohort, pre‐test probability for AD was high (approximately 67%). ROC analysis for pGFAP yielded an AUC of 0.90 (CI 0.85‐0.96). Optimal threshold was 43 pg/mL (77% Sensitivity, 94% Specificity, 97% positive predictive value (PPV)). Additionally, pGFAP levels were marginally higher in AD dementia compared to AD MCI (66.3 [IQR 39.8, 75.3] vs. 42.9 [IQR 31.4, 58.6], *p* = 0.049).

**Conclusion:**

Our results support pGFAP's diagnostic utility in distinguishing AD from non‐AD in routine clinical settings with high AD prevalence, being also consistent with its probable association with disease severity. These findings validate and extend our previous results, suggesting the potential for widespread use of pGFAP to enable earlier, more accurate, and cost‐effective AD detection in clinical practice. However, multicenter studies with larger, diverse cohorts are needed to validate its broader applicability.